# Complete Genome Sequence of a New *Bacteroidaceae* Bacterium Isolated from Anaerobic Biomass Digestion

**DOI:** 10.1128/MRA.01203-19

**Published:** 2019-11-14

**Authors:** Sarah Hahnke, Christian Abendroth, Javier Pascual, Thomas Langer, Francisco M. Codoñer, Patrice Ramm, Michael Klocke, Olaf Luschnig, Manuel Porcar

**Affiliations:** a Leibniz Institute for Agricultural Engineering and Bioeconomy (ATB), Bioengineering Department, Potsdam, Germany; b University of Oldenburg, Department of Human Medicine, Oldenburg, Germany; c Robert Boyle Institut e.V., Jena, Germany; d Technische Universität Dresden, Institute of Waste Management and Circular Economy, Pirna, Germany; e Darwin Bioprospecting Excellence, S.L., Paterna, Valencia, Spain; f Lifesequencing SL-ADM Nutrition, Paterna, Valencia, Spain; g Institute of Agricultural and Urban Ecological Projects affiliated to Berlin Humboldt University (IASP), Berlin, Germany; h Bio H2 Energy GmbH, Jena, Germany; i Institute for Integrative Systems Biology (I2SysBio), University of Valencia–CSIC, Paterna, Valencia, Spain; University of Maryland School of Medicine

## Abstract

Here, we present the genome sequence and annotation of HV4-6-C5C, a bacterial strain isolated from a mesophilic two-stage laboratory-scale leach bed biogas reactor system. Strain HV4-6-C5C may represent a new genus of the family Bacteroidaceae and may have a key role in acidogenesis and acetogenesis steps during anaerobic biomass digestion.

## ANNOUNCEMENT

A new bacterial strain, named HV4-6-C5C, was isolated from a mesophilic laboratory-scale leach bed system using freshly cut grass as the substrate (coordinates 50°51′55.4″N, 11°35′56.1″E). For microbial isolation, BBL Columbia agar (BD Biosciences) supplemented with 5% laked horse blood was applied. DNA was extracted and purified using the Gentra Puregene Yeast/Bact. kit (Qiagen) and the NucleoSpin genomic DNA (gDNA) clean-up kit (Macherey-Nagel). We constructed a Nextera XT library from total genomic DNA and sequenced it using the Illumina NextSeq 500 platform (150-bp paired-end reads). Raw reads were filtered (quality score [*Q*], >20; minimum length, >50 nucleotides [nt]) with BBTools v37.10, yielding 26.42 million paired-end sequences with a mean *Q* value of 33.24. Genome assembly was conducted with SPAdes v3.13.0 (1). A total of 63 contigs with a length of ≥300 nt were obtained, covering a total genome size of 3,869,825 nt and with an estimated GC content of 39.30%. The largest contig was 424,839 nt, and the *N*_50_ value was 183,010 nt.

The assembled sequences were annotated using the NCBI Prokaryotic Genome Annotation Pipeline (2) and the Rapid Annotations using Subsystems Technology toolkit (RASTtk) (3) implemented in the Pathosystems Resource Integration Center (PATRIC) system (4). The genome of strain HV4-6-C5C harbors 2,990 genes, including 2,934 open reading frames, 68 pseudogenes, 3 rRNAs organized in a single operon, 51 tRNAs, and 2 noncoding RNAs (ncRNAs). The genome encodes the enzymes for methylmalonyl-coenzyme A (CoA) mutase (EC 5.4.99.2) and methylmalonyl-CoA epimerase (EC 5.1.99.1), two key enzymes involved in the fermentation of lactate via methylmalonyl-CoA to acetate plus propionate. In addition, the enzymes necessary to perform the conversion of acetyl-CoA to acetate, namely, acetate kinase (EC 2.7.2.1) and phosphate acetyltransferase (EC 2.3.1.8), were also identified. Therefore, strain HV4-6-C5C may have a key role in the acidogenesis and acetogenesis steps during the anaerobic digestion of organic matter.

In order to investigate the taxonomic affiliation of strain HV4-6-C5C, the full-length 16S rRNA gene sequence was compared against the online EzBioCloud database (5). The type strain closest to HV4-6-C5C was Bacteroides stercorirosoris JCM 17103, which shared 94.04% gene sequence similarity with HV4-6-C5C. This result, along with the low average nucleotide identity (6) value between strain HV4-6-C5C and its closest type strains (<95.0%) confirmed that strain HV4-6-C5C is a novel species (7). The average amino acid identity (8) value calculated for strain HV4-6-C5C and B. fragilis NCTC 9343, the type species of the genus Bacteroides, was 67.3%, a value close to the threshold for definition of a new genus (8). However, the percentage of conserved proteins (POCP) (9) and the 16S rRNA gene sequence similarity values for strain HV4-6-C5C and B. fragilis NCTC 9343^T^ were 48.4% and 91.3%, respectively. Both values were lower than the thresholds for the definition of a new genus (9, 10). Hence, we can conclude that strain HV4-6-C5C may represent a new genus within the family Bacteroidaceae.

### Data availability.

Strain HV4-6-C5C was deposited at the German Collection of Microorganisms and Cell Cultures (DSMZ) under the strain designation DSM 104071. This whole-genome shotgun project has been deposited at DDBJ/ENA/GenBank under the accession number VOIU00000000. The version described in this paper is the first version, VOIU01000000. Raw sequence reads are deposited under accession number SRR10177305. The WGS and SRA records are associated with BioProject number PRJNA557591.

## References

[1] Bankevich A, Nurk S, Antipov D, Gurevich AA, Dvorkin M, Kulikov AS, Lesin VM, Nikolenko SI, Pham S, Prjibelski AD, Pyshkin AV, Sirotkin AV, Vyahhi N, Tesler G, Alekseyev MA, Pevzner PA 2012 SPAdes: a new genome assembly algorithm and its applications to single-cell sequencing. J Comput Biol 19:455–477. doi:10.1089/cmb.2012.0021. 22506599 PMC3342519

[2] Tatusova T, DiCuccio M, Badretdin A, Chetvernin V, Nawrocki EP, Zaslavsky L, Lomsadze A, Pruitt KD, Borodovsky M, Ostell J 2016 NCBI Prokaryotic Genome Annotation Pipeline. Nucleic Acids Res 44:6614. doi:10.1093/nar/gkw569. 27342282 PMC5001611

[3] Brettin T, Davis JJ, Disz T, Edwards RA, Gerdes S, Olsen GJ, Olson R, Overbeek R, Parrello B, Pusch GD, Shukla M, Thomason JA 3rd, Stevens R, Vonstein V, Wattam AR, Xia F 2015 RASTtk: a modular and extensible implementation of the RAST algorithm for building custom annotation pipelines and annotating batches of genomes. Sci Rep 5:8365. doi:10.1038/srep08365. 25666585 PMC4322359

[4] Wattam AR, Davis JJ, Assaf R, Boisvert S, Brettin T, Bun C, Conrad N, Dietrich EM, Disz T, Gabbard JL, Gerdes S, Henry CS, Kenyon RW, Machi D, Mao C, Nordberg EK, Olsen GJ, Murphy-Olson DE, Olson R, Overbeek R, Parrello B, Pusch GD, Shukla M, Vonstein V, Warren A, Xia F, Yoo H, Stevens RL 2017 Improvements to PATRIC, the all-bacterial bioinformatics database and analysis resource center. Nucleic Acids Res 45:D535–D542. doi:10.1093/nar/gkw1017. 27899627 PMC5210524

[5] Yoon SH, Ha SM, Kwon S, Lim J, Kim Y, Seo H, Chun J 2017 Introducing EzBioCloud: a taxonomically united database of 16S rRNA and whole genome assemblies. Int J Syst Evol Microbiol 67:1613–1617. doi:10.1099/ijsem.0.001755. 28005526 PMC5563544

[6] Richter M, Rosselló-Móra R, Oliver Glöckner F, Peplies J 2016 JSpeciesWS: a Web server for prokaryotic species circumscription based on pairwise genome comparison. Bioinformatics 32:929–931. doi:10.1093/bioinformatics/btv681. 26576653 PMC5939971

[7] Richter M, Rosselló-Móra R 2009 Shifting the genomic gold standard for the prokaryotic species definition. Proc Natl Acad Sci U S A 106:19126–19131. doi:10.1073/pnas.0906412106. 19855009 PMC2776425

[8] Rodriguez-R LM, Konstantinidis KT 2016 The enveomics collection: a toolbox for specialized analyses of microbial genomes and metagenomes. PeerJ Preprints 4:e1900v1. doi:10.7287/peerj.preprints.1900v1.

[9] Qin QL, Xie BB, Zhang XY, Chen XL, Zhou BC, Zhou J, Oren A, Zhang YZ 2014 A proposed genus boundary for the prokaryotes based on genomic insights. J Bacteriol 196:2210–2215. doi:10.1128/JB.01688-14. 24706738 PMC4054180

[10] Konstantinidis KT, Tiedje JM 2005 Genomic insights that advance the species definition for prokaryotes. Proc Natl Acad Sci U S A 102:2567–2572. doi:10.1073/pnas.0409727102. 15701695 PMC549018

